# Reconstitution of ovarian function following transplantation of primordial germ cells

**DOI:** 10.1038/s41598-017-01648-w

**Published:** 2017-05-03

**Authors:** Ming Zeng, Xiaoyan Sheng, David L. Keefe, Lin Liu

**Affiliations:** 1 State Key Laboratory of Medicinal Chemical Biology, Department of Cell Biology and Genetics, College of Life Sciences, Nankai University, Tianjin, 300071 China; 2 Department of Obstetrics and Gynecology, New York University Langone Medical Center, New York, NY 10016 USA

## Abstract

Ovarian aging occurs earlier than somatic aging. We tested the hypothesis that ovarian functions could be artificially reconstructed by transplantation of primordial germ cells (PGCs). We compared various methods for transplantation of PGCs aggregated with gonadal somatic cells and showed that reconstituted ovaries exhibited folliculogenesis after transplantation of PGCs-aggregates into either kidney capsule or ovarian bursa. Neo-oogenesis occurred early after transplantation, as evidenced by the presence of prophase I meiocytes displaying homologous pairing. Moreover, endocrine function was recovered in ovariectomized recipients, including elevated levels of AMH and estradiol. Interestingly, folliculogenesis in the reconstituted ovaries failed to sustain past four weeks. Regardless of transplantation method, follicles diminished after 45 days, accompanied by increased apoptosis, and were undetectable after two months. Meanwhile, no replicative PGCs or prophase I meiocytes could be found. Together, transplantation of PGCs can effectively reconstitute ovarian functions but for limited time. These data suggest that PGCs do not undergo self-renewal but rapidly enter meiosis following transplantation. Global activation of primordial follicles in artificial ovaries can result in further rapid loss of germ cells. Methods for maintaining self-renewal and expansion *in vivo* of PGCs and controlling follicle activation will be essential for continuing maintenance of the functional reconstructed ovaries.

## Introduction

Ovarian aging precedes somatic aging in most mammalian species. Ovarian aging increases risk of genetic diseases in offspring and contributes to menopause-associated diseases^1^, including cardiovascular mortality and stroke^2, 3^, osteoporosis-related bone fractures^4^, and colorectal cancer^5^. Reconstruction of functional ovaries with restoration of folliculogenesis and hormone secretion have important implications for women who wish to pursue childbearing at midlife, as well as others who have lost ovarian function following chemo- or radiation therapy^6^.

In the mouse, primordial germ cells (PGCs), which give rise to both oocytes and the spermatozoa, are specified at around E7.25. They initiate mitosis and migration, and finally colonize the gonadal ridges at E10.5–11.5^7, 8^. Gonadal ridges differentiate into ovaries or testes according to the sex of embryo^9^. The female PGCs undergo mitotic proliferation and form germ cell cysts^10^. From E17.5, germ cell cysts are broken and oocytes become singly enclosed by several pre-granulosa cells to generate primordial follicles. Primordial follicles provide the source of growing follicles through the entire reproductive life of the female.

Spermatogonia and oogonia differentiate from PGCs and initiate meiosis after E 12.5–13.5 via signaling from the somatic genital ridge^11–13^. PGCs-like cells (PGCLCs) also have been successfully induced from pluripotent stem cells, including ES cells or iPS cells^14, 15^. Moreover, PGCs and PGCLCs complete meiosis *in vitro* and generate functional gametes that produce live offspring^16–18^.

Transplantation of PGCs or male spermatogonia stem cells results in spermatogenesis and restores fertility in sterilized adult mice^19–21^. Functional oocytes also can be developed in the recipients following transplantation or grafting of fetal gonads or PGCs aggregated with fetal somatic pre-granulosa cells into kidney capsule, ovarian bursa or intra-ovarian injection^22–28^. It remains to be determined how long the grafts or transplants continue to generate oocytes and folliculogenesis, and whether transplanted PGCs initiate self-renewal and neo-oogenesis. We undertook series of experiments to investigate for longer-term the function of reconstituted ovaries created by various methods.

## Results

### Folliculogenesis in Recipients Following Transplantation of PGCs

**Figure 1:**
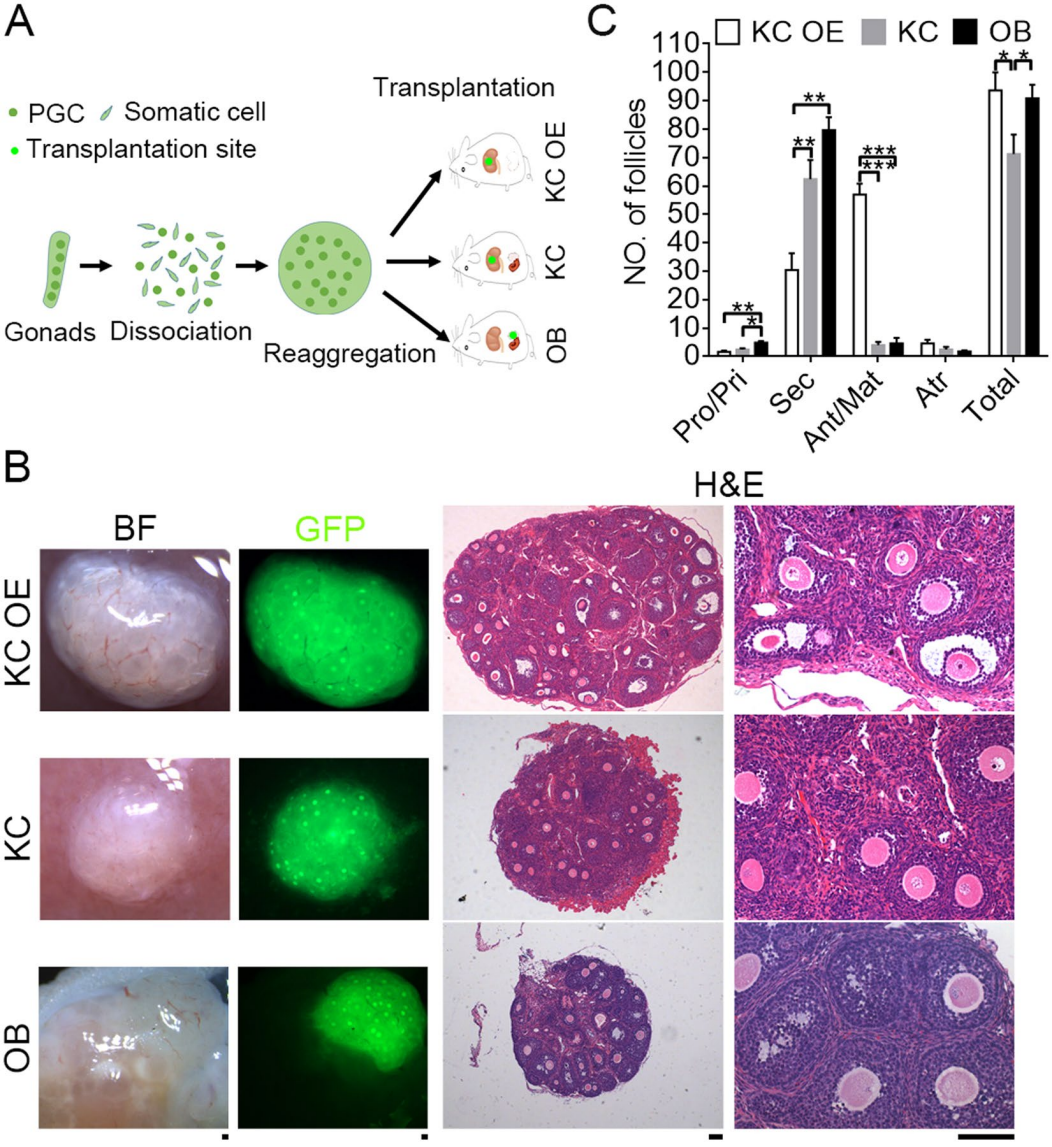
Folliculogenesis in ovary like grafts 28 days following transplantation of PGCs aggregated with gonadal somatic cells. (**A**) Scheme for transplantation procedure and various methods. Female gonads at 12.5-dpc were dissociated, and PGCs aggregated with somatic cells from the same gonad and transplanted under the kidney capsules of bilaterally ovariectomized female recipient mice (KC OE) or the kidney capsules (KC) or the ovarian bursa (OB) of female recipient mice with ovaries. (**B**) Morphology of ovary like grafts 28 days after transplantation using three methods. Follicles in ovary like grafts were readily visible by GFP fluorescence and also shown in sections by H&E staining. Scale bar = 100 μm. (**C**) Number of follicles at various developmental stages in the grafts by different methods of transplantation. Primordial/Primary (Pro/Pri), Secondary (Sec), Antral/Mature (Ant/Mat), Atretic (Atr). Data represents Mean ± SEM (n = 5 mice per group). *P < 0.05; **P < 0.01; ***P < 0.001.

We compared survival, folliculogenesis and oogenesis following transplantation of PGCs-aggregate into locations- kidney capsule, ovarian bursa or ovary. Female gonads at 12.5-dpc were completely dissociated to single cells by enzymatic treatment (Fig. 1A). Dissociated PGCs were aggregated overnight with gonadal somatic/pre-granulosa cells from matched E12.5 fetuses to form single aggregates. Each aggregate contained an average of (1.59 ± 0.28) × 10^5^ PGCs sorted by FACS of SSEA-1 positive cells (n = 3). Efficiency to form oocytes or follicles was low following intra-ovarian injection of dissociated PGCs with gonadal somatic cells labeled with AIE dots or derived from GFP fetuses (Supplementary Fig. S1A–D), consistent with previous studies showing low efficiency in folliculogenesis following transplantation via intro-ovarian injection of E12.5 PGCs with fetal ovarian cells labeled with GFP into adult ovaries^25^.

In subsequent experiments, we collected gonads from β-actin GFP mouse fetuses to distinguish grafted tissues from recipient host cells. To compare ectopic and orthotopic transplantation with respect to formation of the ovary-like graft, we transplanted PGCs aggregated with gonad somatic cells into kidney capsules of ovariectomized (KC OE), kidney capsule (KC) or ovarian bursa (OB) of recipient mice (Fig. 1A). Four weeks following transplantation into kidney capsule or ovarian bursa, PGCs-somatic cell aggregates developed into ovary-like tissue with vascularization on the surface, containing primordial/primary, secondary and antral follicles by bright-field and GFP fluorescence and H&E histology (Fig. 1B). More antral and mature follicles were found in KC OE grafts than in KC and OB grafts (57 ± 3.37 versus 4 ± 1.02 and 4.6 ± 1.69. P < 0.001, Fig.1C). Meanwhile, number of secondary follicles in KC and OB graft was greater than those of KC OE (62.4 ± 6.02 and 79.6 ± 4.12 versus 30.4 ± 5.2, P < 0.01, Fig. 1C). However, more primordial and primary follicles were left in OB graft (4.8 ± 0.52) compared with rare primordial and primary follicles in KC (2.4 ± 4.56, P < 0.05) and KC OE grafts (1.6 ± 0.36, P < 0.01). Follicular atresia was similar among the three graft locations or methods (2.4 ± 0.83, 1.8 ± 0.33 and 4.6 ± 1.08, P > 0.05). Total number of follicles was less in KC graft (71.2 ± 6.11), compared with those of KC OE (93.6 ± 5.71, P < 0.05) and OB grafts (90.8 ± 4.22, P < 0.05). Ovulation and corpus luteum were not observed in any graft 28 days after transplantation, regardless of the transplantation location or method. However, following injection of pregnant mare serum gonadotropin (PMSG), more pre-ovulatory follicles were found in KC OE or OB graft, and even some corpus luteum in OB graft (Supplementary Fig. S2). This also suggests that the grafts can response to gonadotrophin hormone.

These data show that folliculogenesis is achieved in recipients after transplantation of PGCs-aggregates. The majority of PGCs aggregated with gonadal somatic cells undergo folliculogenesis in kidney capsule or ovarian bursa, and removal of the recipient ovaries accelerates folliculogenesis in the graft.

### Neo-Meiosis Occurs in the Reconstituted Ovary-Like Graft

**Figure 2:**
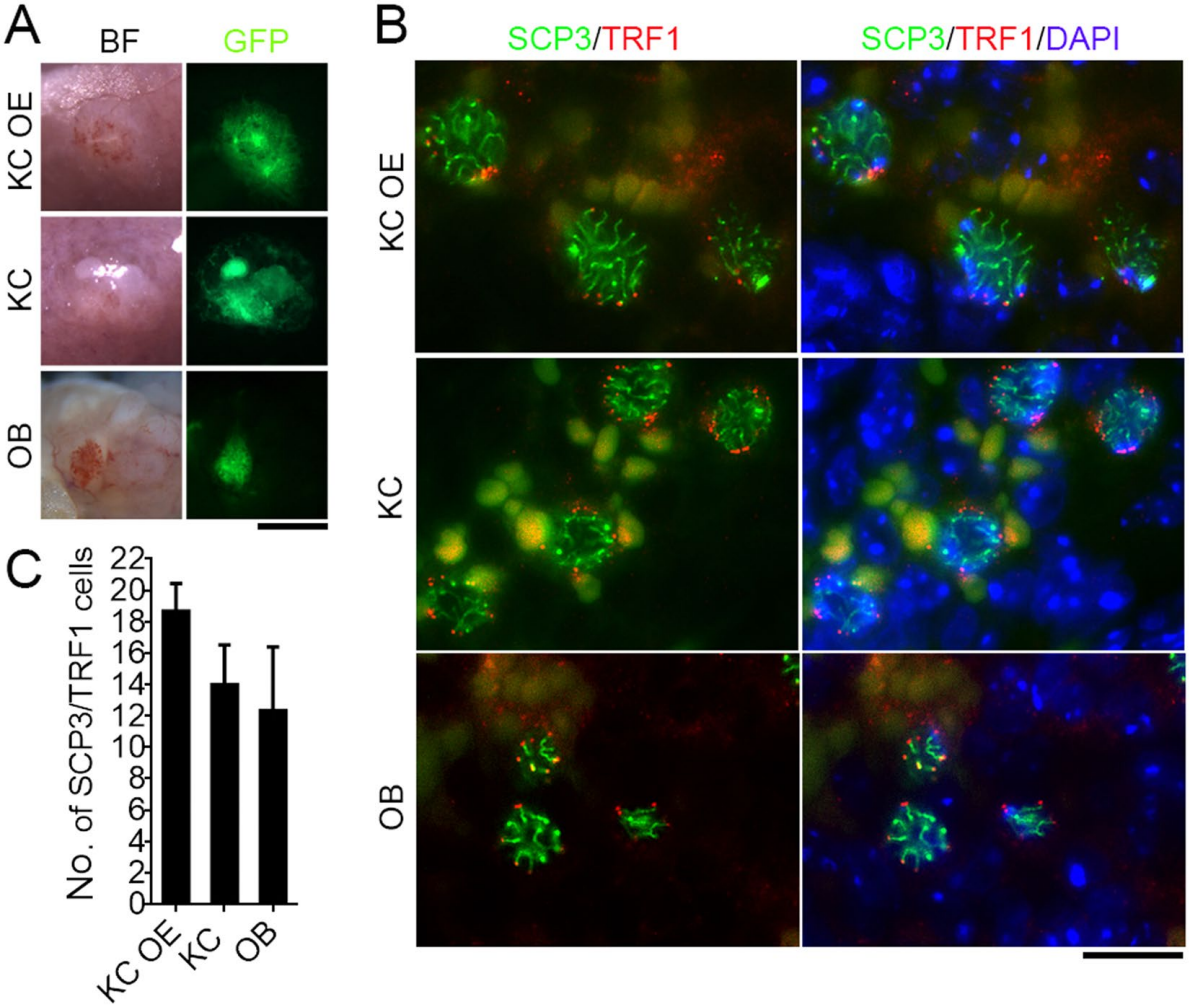
Neo-meiocytes at prophase I in the early grafts 6 days following transplantation of PGCs-aggregates. (**A**) Representative morphology showing the grafts in bright field (BF) and GFP fluorescence. Aggregates were transplanted into kidney capsules of bilaterally ovariectomized mice (KC OE), kidney capsules (KC) or ovarian bursa (OB) of recipient mice with ovaries. Scale bar = 1 mm. (**B**) Co-immunostaining and fluorescence images of SCP3 and TRF1 in grafts, revealing SCP3 lateral filaments and the telomere perinuclear distribution at the termini of the SCP3 filaments in meiocytes at pachytene stage. Scale bar = 10 μm. (**C**) Comparison of the number of prophase I meiocytes with typical perinuclear distribution of telomeres revealed by TRF1 and distinct SCP3 lateral filaments. Data represents Mean ± SEM (n = 3 sections per graft).

Next, we asked whether neo-meiosis proceeds appropriately in recipient mice following transplantation of PGCs. Typical telomere bouquet clustering with perinuclear distribution, associated with homologous bivalents identified by synaptonemal complex protein 3 (SCP3) elements are essential for homologous chromosome pairing and synapsis and serve as a definitive marker of meiocytes at prophase I^29–33^. By co-immunostaining and fluorescence microscopy of SCP3 with the telomere-associated protein TRF1, we validated the presence of the meiocytes in 16.5-dpc fetal ovaries as evidenced by the typical perinuclear distribution of TRF1 foci at telomeres connecting distinct SCP3 lateral filaments. Six days following transplantation of PGCs-aggregates, grafts were readily identified by GFP fluorescence (Fig. 2A). The KC OE, KC or OB grafts possessed early meiocytes displaying perinuclear distribution of telomeric TRF1 foci associated with the termini of distinct SCP3 lateral filaments, indicative of pachytene stage (Fig. 2B). Some early meiocytes showed perinuclear distribution of TRF1 foci and bouquet formation at leptotene–zygotene transition in KC and OB grafts. An average of 18.67 ± 1.44, 14 ± 2.05 and 12.33 ± 3.31 early meiocytes/section was estimated in KC OE, KC and OB graft respectively (Fig. 2C). PGCs in KC OE graft might enter meiosis faster than did KC and OB grafts, consistent with more antral and mature follicles in KC OE graft and mostly secondary follicles in KC and OB grafts 28 days after transplantation. Moreover, meiocytes at diplotene stage as evidenced by the presence of SCP3 laternal elements and minimal or no central SCP1 elements were also found in the graft sections (Supplementary Fig. S3).

Taken together, all three methods resulted in neo-meiosis. Meiocytes from PGCs appeared shortly after transplantation. The developmental stage of these meiocytes was mostly at pachytene and diplotene equivalent to those of the 16.5-dpc fetal ovary.

### Evaluation of long-term folliculogenesis in ovary-like grafts

**Figure 3:**
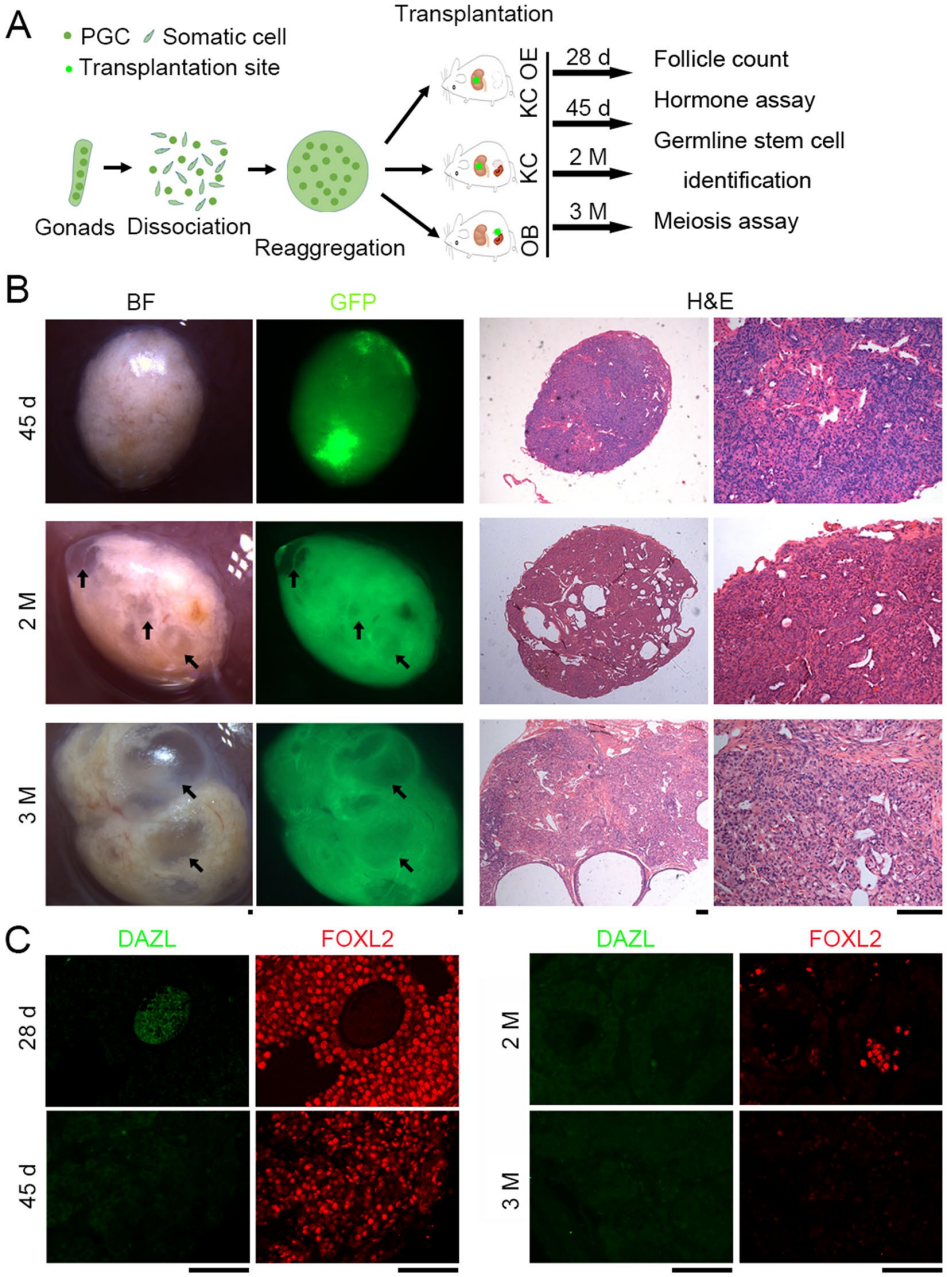
Loss of folliculogenesis 45 days following transplantation of PGCs- aggregates. (**A**) Experimental design for longer-term transplantation. Female gonads at 12.5-dpc were dissociated, and PGCs were aggregated with somatic cells from the same gonad and transplanted using various methods shown above. The grafts were retrieved 28 days, 45 days, 2 months and 3 months respectively after transplantation and subjected to the functional analysis as indicated. (**B**) Morphology of ovary like grafts collected at 45 days, 2 months and 3 months after transplantation by KC OE (left). Grafts collected at 28 days are similar to those shown in Fig. 1B KC OE. Follicles were undetectable in the sections by H&E staining following transplantation for longer-term (45 days, 2 months and 3 months) (right). Arrows indicate large vacuoles without follicles verified by H&E histology. (**C**) Co-immunostaining and fluorescence of residual cytoplasmic DAZL in oocytes and FOXL2 a specific marker for granulosa cells in grafts. Scale bar = 100 μm.

We collected reconstituted ovarian grafts at various time points (28 days, 45 days, 2 and 3 months) following transplantation and analyzed them for evidence of ovarian function (Fig. 3A). Consistently, OE grafts 28 days after transplantation contained many antral and mature follicles in the KC. However, by 45 days and 2 months after transplantation no follicles were found in KC OE grafts. Instead, grafts contained corpus albicans, residues of degraded corpus lutei, and many vacuoles or cysts without follicles, as revealed by GFP fluorescence and H&E histology (indicated by arrow, Fig. 3B; Table 1). More corpus albicans and large cysts/vacuoles were present in grafts with weaker GFP fluorescence three months after KC OE transplantation (arrow, Fig. 3B). Thus, follicles were exhausted in grafts within 45 days following transplantation of PGCs (Table 1).

**Table 1:** Folliculogenesis of PGCs-aggregates transplanted into the recipient mice.

Methods	Time	No.		
		Mice transplanted	Retrieved grafts (%)	With Follicles (%)
KC OE	28 days	9	9 (100)	9 (100)
	45 days	3	3 (100)	0
	2 months	6	5 (83.3)	0
	3 months	5	4 (80)	0
KC	28 days	10	9 (90)	9 (100)
	45 days	3	3 (100)	0
	2 months	7	6 (85.7)	0
	3 months	5	4 (80)	0
OB	28 days	13	7 (53.8)	5 (71.4)
	45 days	6	4 (66.7)	0
	2 months	7	4 (57.1)	0
	3 months	7	4 (57.1)	0

One aggregate was transplanted into one recipient mouse by KC OE (Kidney capsule ovariectomized), KC (Kidney capsule) or OB (Ovarian bursa). Time, days or months as indicated following transplantation of the PGCs-aggregates.

Similarly, size of KC and OB grafts was decreased and follicles were only rarely found in reconstituted ovaries by 45 days, 2 and 3 months after transplantation and albicans and vacuoles were frequently found in these grafts (Supplementary Figs S4 and S5; Table 1).

To trace the fate of germ cells during folliculogenesis, we performed immunofluorescence of the germ cell marker DAZL^34^ and the granulosa cell marker FOXL2 in sections of the grafts. In fetal ovaries, FOXL2 positive pre-granulosa cells surrounded and connected germ cell cysts (DAZL positive) in support of follicle activation and development. Residual cytoplasmic DAZL positive oocyte surrounded by layers of FOXL2 positive granulosa cells constituted most antral and mature follicles in the KC OE grafts 28 days after transplantation (Fig. 3C), supporting the histology data showing robust folliculogenesis in reconstituted ovaries. Meanwhile, many FOXL2 positive granulosa cells also resided in the stroma of the graft. However, no DAZL positive cells with FOXL2 granulosa cells were found, and the number of FOXL2 positive granulosa cells decreased in grafts by 45 days, and even more sharply two and three months after transplantation by KC OE, KC, or OB (Supplementary Figs S4 and S5). Fluorescence signal intensity of FOXL2 was much lower suggestive of degradation of granulosa cells three months following transplantation. These data suggest that loss of germ cells and of folliculogenesis takes place by 45 days or earlier after transplantation.

### Follicle Atresia Associated with Apoptosis in the Grafts

We performed TUNEL assays to detect apoptosis in grafts 28 days, 45 days, 2 and 3 months after transplantation. Fragmented nuclei showing positive TUNEL signals (green) were defined as apoptotic cells. Few apoptotic cells per section (4.67 ± 1.78) were found in the KC OE graft after 28 days, significantly lower than those of 45 days following transplantation of PGCs (23 ± 3.38, P < 0.5). Also, fewer apoptotic cells were found in grafts collected after 2 months (6.33 ± 1.78, P < 0.5) and even fewer in grafts of 3 months (0.33 ± 0.27) than those of 45 days after transplantation (Supplementary Fig. S6A, S6B). Similarly, significantly more apoptotic cells were found in KC or OB grafts after 45 days compared to 28 days, 2 and 3 months after transplantation (Supplementary Fig. S6A, S6B). Furthermore, follicular atresia, as shown by degenerative oocytes and increased levels of apoptosis in the granulosa cells of the grafts, was visible even earlier- 32 days after transplantation (Supplementary Fig. S7).

To determine endocrine function resulting from transplantation of PGCs, we measured the serum levels of follicle-stimulating hormone (FSH), estradiol (E2) and anti-müllerian hormone (AMH) in host animals. Serum FSH levels were reduced and levels of E2 and AMH elevated in recipients with KC OE grafts, compared to controls without grafts 28 days after transplantation (Supplementary Fig. S8A). Notably, E2 and AMH levels decreased with increasing time after transplantation and reached a nadir by three months post transplantation, comparable to controls, consistent with the loss of follicles and ovarian reserve shown earlier. In the presence of host ovaries, the KC or OB grafts did not change serum levels of E2 and AMH, similar to controls (Supplementary Fig. S8B, S8C). The host ovaries minimize the endocrine function of grafts following transplantation.

These findings demonstrate that ovary-like grafts, generated from PGCs aggregated with gonadal somatic cells and transplanted into the kidney capsules of ovariectomized mice, show endocrine function. Loss of follicles associated with follicle atresia and increased apoptosis of grafts by 45 days or perhaps as early as 32 days after transplantation contribute to diminished endocrine function.

### Presumptive Germline Stem Cells (GSCs) in Reconstituted Ovaries

**Figure 4:**
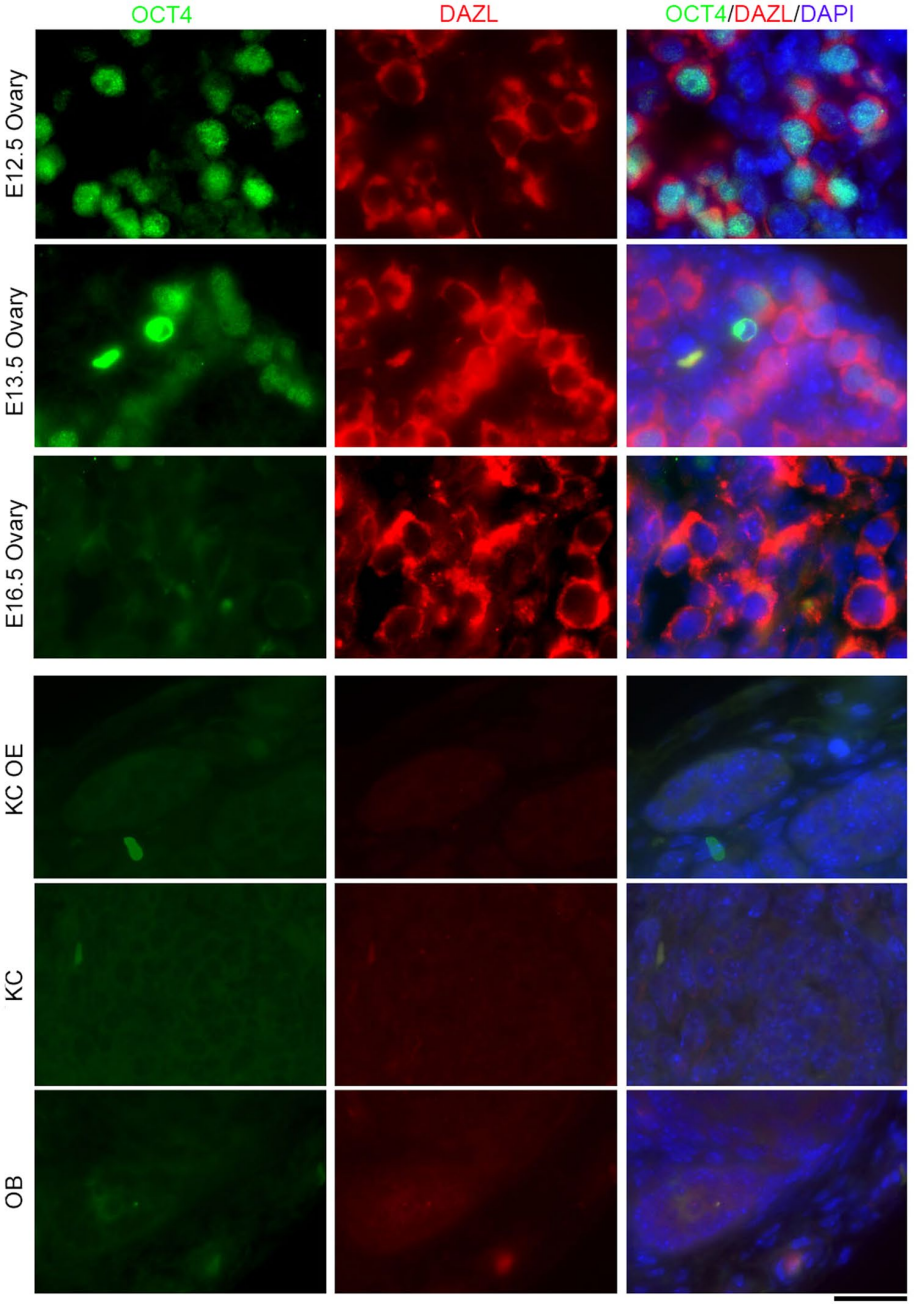
Presumptive germline stem cells (GSCs) are found in fetal ovaries but not in grafts 28 days after transplantation of PGCs-aggregates. Co-immunostaining and fluorescence of nuclear OCT4 and germ cell marker cytoplasmic DAZL in E12.5, E13.5 and E16.5 fetal ovaries (upper), and in grafts (bottom) 28 days following transplantation by different methods (Kidney capsule with ovariectomized, Kidney capsule and ovarian bursa). Scale bar = 20 μm.

To search for presumptive GSCs in grafts, we performed immunofluorescence microscopy to reveal co-expression of OCT4 and DAZL. In E12.5 fetal ovaries served as positive controls, most PGCs/GSCs stained strongly positive for nuclear OCT4 with cytoplasmic DAZL fluorescence. Only weak nuclear OCT4 staining appeared in E13.5 ovaries entering meiosis. By E16.5, when most meiocytes are at pachytene stage, OCT4 expression was completely lost (Fig. 4). Cells of size similar to E12.5 PGCs/GSCs, characterized by cytoplasmic DAZL and nuclear OCT4 expression, were not found in reconstituted grafts after 28 days, irrespective of transplantation location or methods (Fig. 4, bottom). Cells with nuclear OCT4 and cytoplasmic DAZL also were not detectable in grafts 2 and 3 months post transplantation (Supplementary Fig. S9).

**Figure 5:**
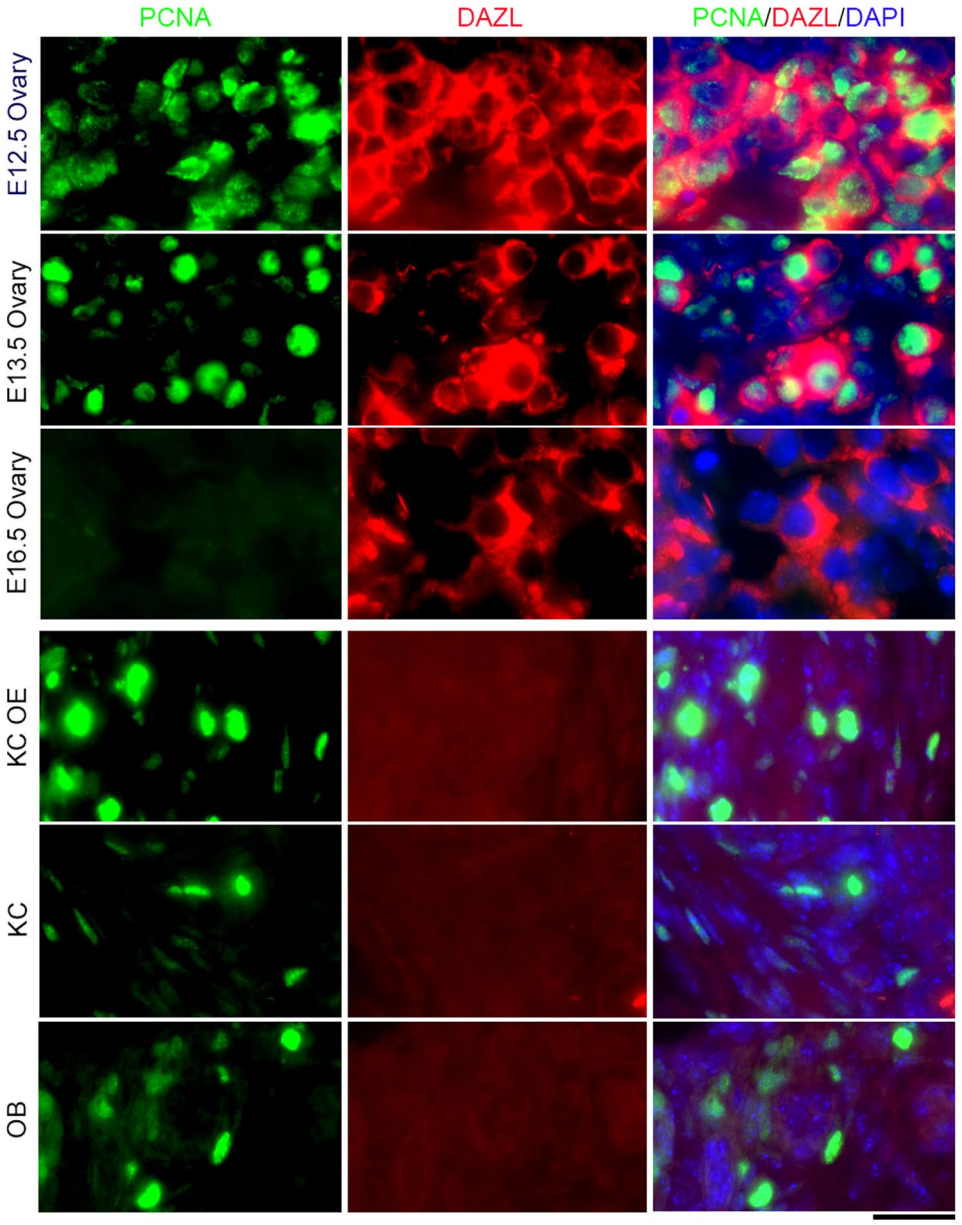
Loss of proliferative germ cells in grafts 28 days following transplantation of PGCs-aggregates. Co-immunostaining of proliferative marker PCNA and germ cell marker cytoplasmic DAZL in E12.5, E13.5 and E16.5 fetal ovaries (upper), and in grafts (bottom) by different methods of transplantation. Scale bar = 20 μm.

To identify mitotically active, proliferating GSCs, we performed co-immunostaining of the proliferative marker PCNA and DAZL in all grafts, and compared them to fetal ovaries. Many PGCs were strongly positive for nuclear PCNA in E12.5 and E13.5 fetal ovaries, but not in E16.5 ovaries with pachytene meiocytes (Fig. 5). Cells positive for nuclear PCNA but negative for cytoplasmic DAZL often were found on the edges or in the middle of the graft 28 days after transplantation (Fig. 5, bottom). Presumably these cells are granulosa cells or other somatic cell types, as previously reported^33^. In addition, oocytes were weakly stained for cytoplasmic DAZL, surrounded by strong PCNA staining in granulosa cells within follicles of the grafts (Supplementary Fig. S10A). Cells with positive nuclear PCNA and cytoplasmic DAZL staining were not observed in grafts 2 or 3 months after transplantation. PCNA positive cells were rarely found in grafts 2 and 3 months after KC OE, KC and OB transplantation (Supplementary Fig. S10B, S10C), indicating that proliferative PGCs wane gradually in grafts over the longer-term. Additionally, neo-meiocytes, shown as SCP3 lateral filaments and perinuclear distribution of TRF1 foci, were not detectable in grafts 28 days, 2 and 3 months after transplantation (Supplementary Fig. S11).

Absence of proliferative GSCs and neo-meiosis in grafts provides an explanation for the follicular exhaustion- follicles undergo atresia by apoptosis, and replenishment of new follicles does not occur 32–45 days following transplantation.

## Discussion

We confirm that transplantation of PGCs/GSCs aggregated with gonadal somatic cells effectively restores folliculogenesis and endocrine function, and transplantation to either kidney capsule or ovarian bursa results in similar effectiveness in recipient mice with or without ovaries. Furthermore, we find that the majority of PGCs enter meiosis and undergo folliculogenesis soon after transplantation, regardless of the location of the graft, and ovarian vasectomy accelerates follicular development and maturation. By four weeks following transplantation, however, proliferative PGCs and neo-meiocytes have disappeared. Instead, increased apoptosis occurs by 45 days or earlier following transplantation by the various methods.

The presence of the host ovary may prevent follicle further development to antral and mature stage, as more secondary but fewer antral or mature follicles are found in ovary-like grafts by kidney capsule and ovarian bursa transplantation when host ovaries remain, whereas more antral and mature, and fewer secondary follicles exist in grafts formed by KC OE. Rare primordial and primary follicles exist in all grafts at 28 days following transplantation by KC OE, KC and OB. We anticipated restoration of ovarian function, with persistent folliculogenesis and hormone secretion following transplantation of PGCs aggregated with fetal gonadal somatic cells. Unexpectedly, the PGCs fail to self-renew and follicles degenerate, along with decreased hormone secretion in host animals after four weeks. Because all three transplantation methods produced similar outcomes, it is unlikely that the niche provided by adult ovaries or kidneys is conducive to PGC survival. Indeed, mounting evidence demonstrates that adult female mice neither require nor contain active germ-line stem cells or produce new oocytes *in vivo* ^25, 35^. Consistently, neo-meiocytes exhibiting homologous pairing by SCP3 filaments with the termini associated with telomeres at pachytene stage are found only within a few days following transplantation of PGCs, but not after 28 days or earlier. In line with this, most PGCs are simultaneously activated to enter meiosis, such that primordial and primary follicles rarely reside in grafts 28 days after transplantation. It is likely that PGCs undergo oogenesis in a synchronous single wave^14, 27^. Small molecule, such as AS101^36^, may partially stall primordial follicle activation and follicle loss.

Without a continuous supply of PGCs and neo-meiosis, oocytes and follicles eventually are depleted from the graft. Oocytes influence granulosa cell development primarily by secretion of paracrine factors^37^. We find that apoptosis associated with follicle atresia is markedly increased in reconstituted ovaries at 32 to 45 days after transplantation, coincident with reduction in proliferation and number of granulosa cells as revealed by immunofluorescence of PCNA and FOXL2. The follicular microenvironment also could deteriorate in reconstituted ovaries over time, and contribute to reduced follicle numbers. Granulosa cells and cumulus cells are intimately connected with oocytes, and play critical roles in oocyte growth and follicular development^37–39^.

Together, functional ovaries can be reconstituted by transplantation of PGCs into recipients, but the viability of these grafts carries a time stamp. Methods for maintaining primordial/primary follicle pools and of delaying folliculogenesis will be essential for long-term maintenance of the reconstituted ovaries, potentially useful in mitigating ovarian aging.

## Methods

### Mice and Care

Animals were cared for and treated according to guidelines set by U.S. National Research Council, and the use of mice for this research was approved by the Nankai University Animal Care and Use Committee. C57BL/6NCrSlc (B6) females were mated with C57BL/6NCrSlc or green fluorescent protein (GFP)-expressing transgenic C57BL/6-Tg (CAG-EGFP) C14-Y01-FM131Osb males (obtained from Model Animal Research Center of Nanjing University) to obtain B6 or B6-GFP fetal ovaries of 12.5-dpc, as judged by the presence of a copulation plug (0.5-dpc).

### PGCs and Transplantation

Collection of PGCs from C57BL/6-GFP fetal ovaries and re-aggregation of PGCs with somatic cells from the same gonads were performed according to the protocol described^27^, with minor modifications. Briefly, gonads were dissociated into single cells and aggregated with Phytohemagglutinin-P. Then the cells were centrifuged as an aggregate and cultured overnight.

For transplantation, 8–12 weeks old B6 recipient females were randomly divided into three groups as follows: Group 1: Each aggregate containing (1.59 ± 0.28) × 10^5^ PGCs and (5.95 ± 0.46) × 10^5^ somatic cells was transplanted beneath the kidney capsule (abbreviated as KC) of a recipient mouse; Group 2: Each aggregate was transplanted beneath the kidney capsule of a bilaterally ovariectomized recipient mouse (KC OE); Group 3: Each aggregate was transplanted beneath the ovarian bursa (OB) of a recipient mouse.

Kidney capsule transplantation was performed based on the methods described^26, 27^. Briefly, one aggregate was implanted in the “pocket” which was made between the kidney capsule and kidney tissue. Ovarian bursa transplantation was performed based on the reports^22, 40^. One aggregate was quickly placed into the incision made in the ovary under the ovarian bursa. Shamed operation was performed but without transplantation served as controls for each group. The transplantation procedures were completed in 5 minutes for each mouse.

Intra-ovarian injection procedure was performed according to the protocol described^25^. Gonads were dissociated into cell suspension and injected to one ovary of mouse by a bevel glass pipette. More details are provided in Supplementary Methods.

### Retrieval of the Grafts/Reconstituted Ovaries and Immunofluorescence Microscopy

At various days following transplantation (6 days, 28 days, 32 days, 45 days, 2 months, and 3 months), the grafts were carefully retrieved from the kidney capsules or ovarian bursa of recipient mice and kidney or original ovarian tissues removed as much as possible under the epi-fluorescence microscope. Grafts were fixed for 16–24 h in 4% paraformaldehyde and embedded in paraffin. Embedded samples were sectioned (5 μm) and subjected to hematoxylin-eosin (HE) staining and immunofluorescence.

### Statistical Analysis

Data are represented as mean ± standard error of mean (SEM). Statistical significance was determined using the unpaired Student’s t-Test with 2-tailed distribution of unequal variance. Differences were considered to be statistically significant for P < 0.05 (*), P < 0.01 (**), or P < 0.001 (***).

## Electronic supplementary material

Supplementary methods and figures
