# Supplementary material for: Reconstitution of ovarian function following transplantation of primordial germ cells
Source: Sci Rep. 2017 May 3;7:1427. doi: 10.1038/s41598-017-01648-w (PMC5431110; doi:10.1038/s41598-017-01648-w)
Supplement: Supplementary file 1 — Supplementary methods and figures [file 41598_2017_1648_MOESM1_ESM.pdf]

Supplementary Information

Reconstitution of ovarian function following transplantation of primordial germ cells

Ming Zeng, Xiaoyan Sheng, David L. Keefe, and Lin Liu

Supplementary Methods

PGCs and Transplantation.

Collection of PGCs from C57/BL6-GFP fetal ovaries and re-aggregation with somatic cells from the same gonads was performed according to the protocol described [1], with minor modifications. Briefly, eight 12.5-dpc ovaries without attached mesonephroses were mixed in a 1.5 ml centrifuge tube and dispersed into single cells in a 100 µl solution of 0.25% trypsin-EDTA plus 0.2% collagenase IV at 37°C for 15 min. After the addition of medium 199 supplemented with 10% FBS, 1% antibiotics (penicillin-streptomycin) and 1 mM L-glutamine, and pipetted 5–10 times, cells were resuspended in 100 µl of culture medium. Phytohemagglutinin-P (Sigma) was added to a final concentration of 35 µg/ml and the mixture of cells was incubated at 37°C for 10 min. Samples were centrifuged at 9000 g for 10 s and centrifuged again for 30 s after 180° rotation. Pellets of re-aggregated cells were gently removed from the tubes with a truncated 200 µl micropipette and the aggregates were cultured 12-18 h at 37°C in culture medium in 96-well plate coated with 2% agarose in PBS.

Kidney capsule transplantation was performed based on the methods described [1,2]. Briefly, one recipient mouse was intraperitoneally anesthetized with 2,2,2-Tribro-moethanol and a small "pocket" was made between the kidney capsule and kidney tissue. One aggregate was picked up with a mouth-controlled glass Pasteur pipette and implanted in the "pocket". Ovarian bursa transplantation was performed based on the reports [3,4]. Briefly, a hole was pierced in the ovarian bursa and an incision made in the ovary of the animal. A glass capillary aspirated with one aggregate was inserted into ovarian bursa through the hole and the aggregate quickly placed into the incision of the ovary. Shamed operation was performed but without transplantation served as controls for each group. The transplantation procedures were completed in 5 minutes for each mouse.

For intra-ovarian injection, cell suspension from eight B6-GFP 12.5-dpc ovaries were filtered through a 40-µm pore cell strainer to avoid cell aggregations and resuspended in 10µl cold culture medium. The injection procedure was performed according to the protocol described [5]. Briefly, fetal ovarian cells were injected into one of the ovaries (3.5×10^5 cells/5 µl per point, two points per ovary), and culture medium was injected into the opposite ovary as the negative control. Microinjection of each ovary was performed using a glass pipette with a 45-µm tip. When ovaries

from B6 without GFP were used as donor for limited experiments initially on intra-ovarian injection, single cells from 12.5-dpc ovaries was exposed to 1 nM AIE dots for 20 min at 37°C and washed twice with culture medium [6].

Immunofluorescence Microscopy.

For immunofluorescence, after deparaffinizing, rehydrating, and washing in 0.01 M PBS (pH 7.2–7.4), sections were subjected to high-pressure antigen recovery sequentially in 0.01% citrate buffer (pH 6.0) for 3 min, permeabilized in 0.2% Triton X-100 for 30 min, blocked with 5% goat serum and 0.1% BSA in PBS for 2 h at room temperature, and then incubated with the primary antibodies overnight at 4°C. Blocking solution without the primary antibody served as negative control. After washing with PBS, sections were incubated with appropriate secondary antibodies (Alexa Fluor 594 or 488, Invitrogen). The sections were then stained with 1 μg/ml DAPI for 15 min to reveal nuclei, washed, mounted in Vectashield (Vector Laboratories), and photographed with a Zeiss Axio Imager Z1 (Carl Zeiss). The following primary antibodies were used for immunofluorescence: DAZL (ab34139, Abcam, 1: 400), VASA (ab13840, Abcam, 1: 500), SCP3 (NB 300-232, Novus Biologicals, 1: 400), PCNA (SC25280, Santa Cruz, 1:400), TRF1 (TRF12-S, Alpha Diagnostics, 1:2000), SCP3 (ab97672, Abcam, 1: 400), Oct3/4 (SC-5279, Santa Cruz, 1:200), GFP (AG281, Beyotime Biotechnology, 1:200), Foxl2 (ab5096, Abcam, 1:250).

Follicle Count.

The grafts were carefully retrieved and subsequently dehydrated with graded alcohols, cleared in xylene, and embedded in paraffin wax. The serial sections (5 μm) from each graft were aligned in order on glass microscope slides, stained with hematoxylin and eosin Y (H&E) and analyzed for the number of follicles at four different developmental stages in every fifth section with random start in the first five sections. The total number of follicles per graft was calculated by combining the counts of every fifth section throughout the whole graft [7].

The follicles were categorized into primordial or primary, secondary and antral or mature and atretic accordingly [7,8]. Primordial, primary, and intermediate-stage follicles were identified by the presence of an oocyte surrounded by a single layer of flat, squamous, or cuboidal cells. Secondary follicles were characterized as having more than one layer of granulosa cells with no visible antrum. Antral or mature follicles possessed small areas of follicular fluid (antrum) or a single large antral space. Only those follicles containing an oocyte with a clearly visible nucleus were scored.

Identification of Homologous Pairing in Early Meiocytes.

E16.5 ovaries and grafts collected 6 days after transplantation were continuously sectioned as described above. Three sections per graft were randomly chosen for co-immunostaining of SCP3 with the TRF1. Number of cells that have typical perinuclear distribution of TRF1 and distinct SCP3 lateral filaments were counted.

Apoptosis by TUNEL Assay.
The apoptosis of tissue sections in the graft was revealed by catalytically incorporating fluorescein-12-dUTP at 3′-OH DNA ends with the Terminal Deoxynucleotidyl Transferase, Recombinant, enzyme (rTdT) using a commercial kit (TB235, Promega). Briefly, after deparaffinizing, rehydrating, washing in PBS, fixing in 4% paraformaldehyde and treatment with proteinase K treatment, sections were incubated with rTdT buffer at 37°C for one hour. Sections were stained with 1 μg/ml DAPI for 15 min, washed, mounted in Vectashield, and immediately analyzed under a fluorescence microscope using a standard fluorescein filter set. Deionized water instead of rTdT in buffer served as negative control.

Three sections per graft at every 20 section intervals were subject to apoptosis analysis. Total number of TUNEL positive cells was calculated by combining the counts of three sections in each graft.

Hormone Assays.
Serum follicle-stimulating hormone (FSH) and estradiol (E2) levels were determined by direct radioimmunoassay using commercial kits (B03PZB and B05PZB, China Diagnostics Medical Corporation). Anti-müllerian hormone (AMH) levels were assayed by ELISA kit (CK-E90200, Hangzhou EastBiopharm CO., LTD). Quality control serum, sterilized distilled water, and five series diluted standard samples for a standard curve were tested for each serum sample. The intra- and inter-assay coefficients of variability for AMH, FSH, and E2 were below 8% and 12%.

1. Matoba, S. & Ogura, A. Generation of functional oocytes and spermatids from fetal primordial germ cells after ectopic transplantation in adult mice. *Biol Reprod* 84, 631-638 (2011).
2. Qing, T. *et al.* Mature oocytes derived from purified mouse fetal germ cells. *Hum Reprod* 23, 54-61 (2008).
3. Hashimoto, K., Noguchi, M. & Nakatsuj, N. Mouse Offspring Derived from Fetal Ovaries or Reaggregates Which were Cultured and Transplanted into Adult Females. . *Develop Growth & Differ* 34, 233-238 (1992).
4. Hayashi, K. & Saitou, M. Generation of eggs from mouse embryonic stem cells and induced pluripotent stem cells. *Nat Protoc* 8, 1513-1524 (2013).
5. Zhang, H. *et al.* Experimental evidence showing that no mitotically active female germline progenitors exist in postnatal mouse ovaries. *Proc Natl Acad Sci U S A* 109, 12580-12585 (2012).
6. Ding, D. *et al.* Precise and Long-Term Tracking of Adipose-Derived Stem Cells and Their Regenerative Capacity via Superb Bright and Stable Organic Nanodots. *Acs Nano* 8, 12620-12631 (2014).
7. Liu, M. *et al.* Resveratrol protects against age-associated infertility in mice. *Hum Reprod* 28, 707-717 (2013).
8. Myers, M., Britt, K. L., Wreford, N. G. M., Ebling, F. J. P. & Kerr, J. B. Methods

for quantifying follicular numbers within the mouse ovary. *Reproduction* 127, 569-580 (2004).

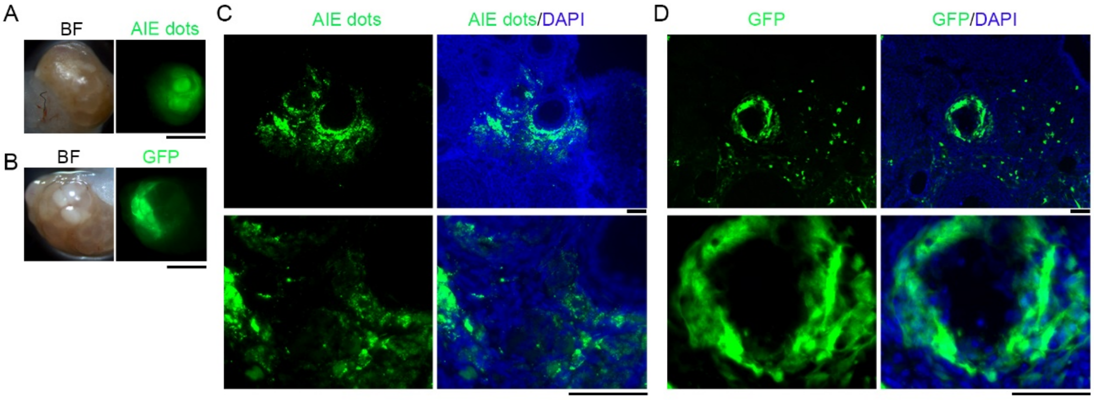

Fig. S1. PGCs and gonadal somatic cells labeled with AIE dots or GFP following intra-ovarian injection. (*A*) Fluorescence of gonad cells labeled with AIE dots 28 days after intra-ovarian injection. (*B*) Single cells dissociated from female E12.5 gonads from B6-GFP fetuses injected into ovary and exhibit GFP fluorescence 28 days after injection. (*C*) Gonad cells labeled with AIE dots are scattered in ovarian stroma and rarely in oocytes of follicles. Some are in the area of granulosa cells. (*D*) Gonad cells with GFP fluorescence were scattered in ovarian stroma and rarely in oocytes of follicles. Some are in the area of granulosa cells. Scale bar =1 mm (*A*, *B*) and 50 μm (*C* and *D*).

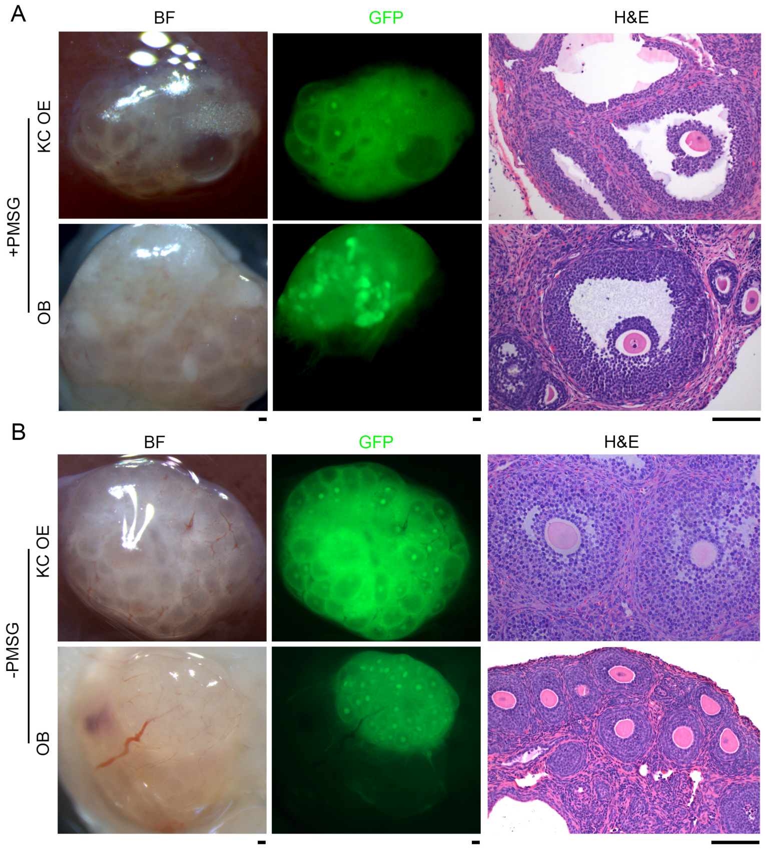

Fig S2. Representative morphology of OB and KC OE grafts 28 days after transplantation. BF, bright-field. 5 IU PMSG was intraperitoneally injected to each recipient 48 hours before graft collection (*A*, +PMSG; *B*, -PMSG served as control). Scale bar = 100 μm.

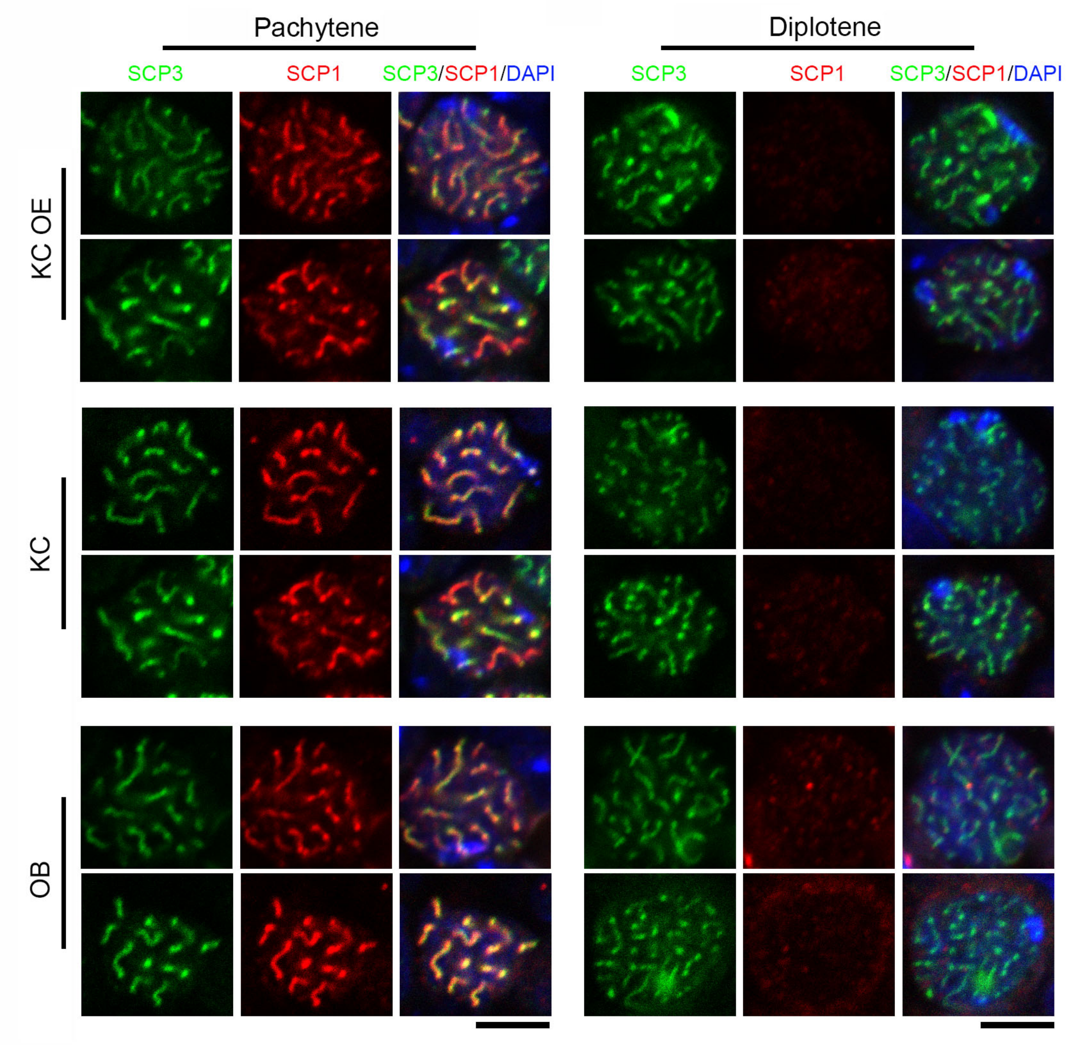

Fig. S3. Representative images of pachytene and diplotene meiocytes in the KC OE, KC and OB graft 6 days after transplantation, identified by co-staining of SCP3 and SCP1 antibodies. Scale bar = 5 µm.

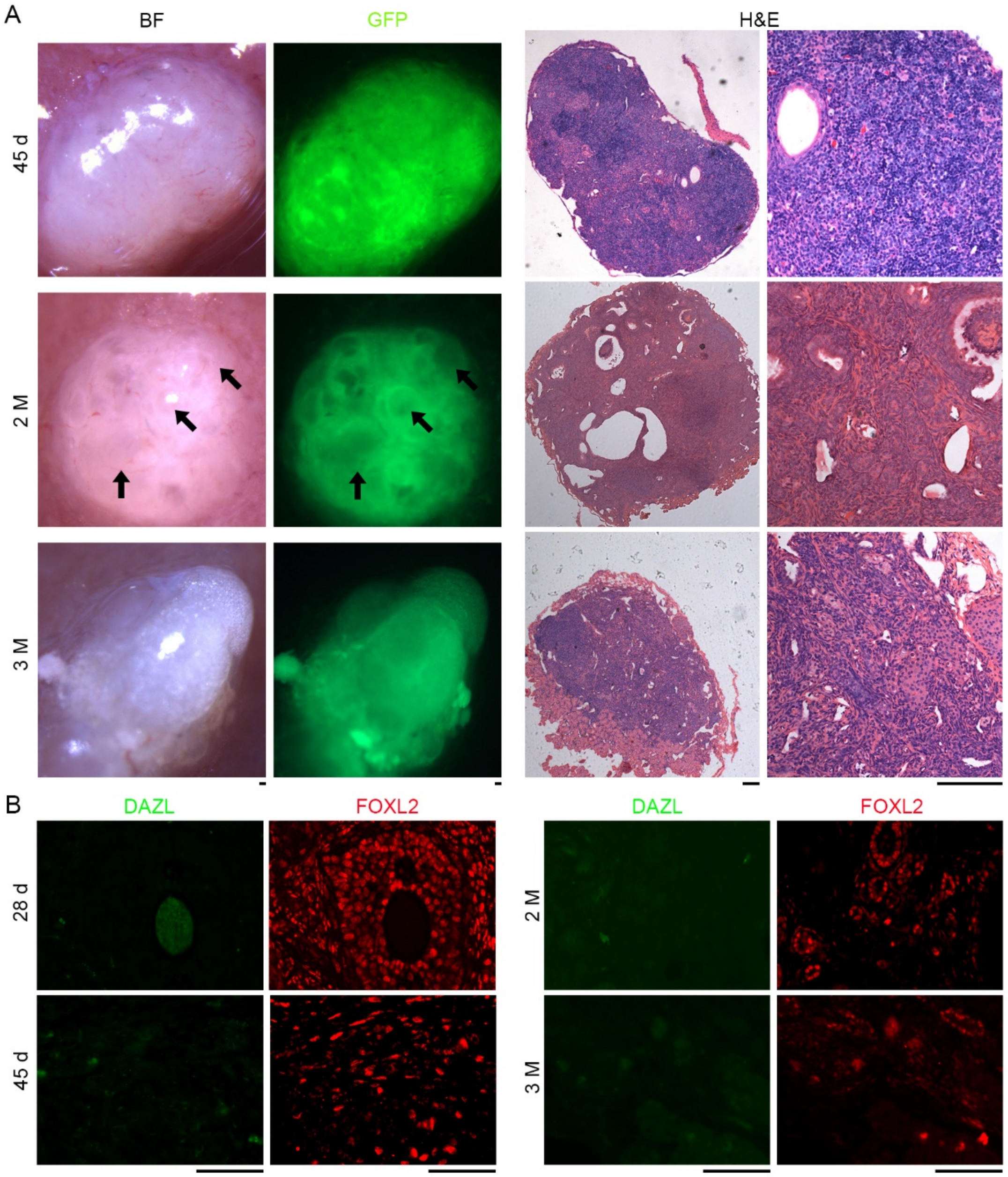

Fig. S4. Loss of folliculogenesis in the graft 45 days following transplantation into kidney capsule (KC) of PGCs aggregated with gonadal somatic cells. (*A*) Morphology of ovary like grafts at 45 days, 2 months and 3 months after transplantation (left). Follicles are not detectable in the sections by H&E staining following transplantation for longer-term (45 days, 2 months and 3 months) (right). The arrows indicate large vacuoles without follicles. (*B*) Co-immunostaining and fluorescence of DAZL and granulosa cell marker FOXL2. Scale bar =100 µm.

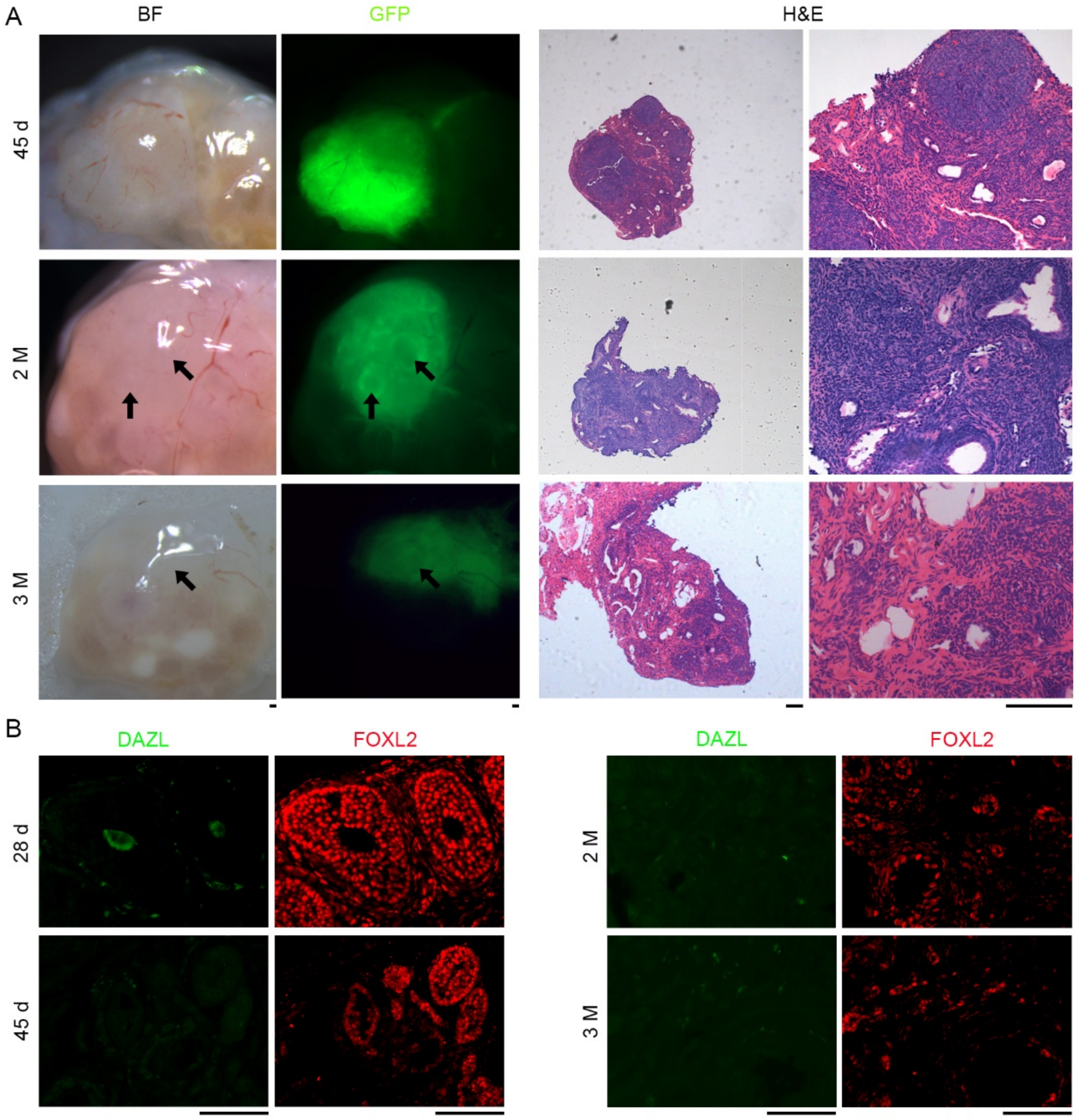

Fig. S5. Loss of folliculogenesis in the graft 45 days following transplantation into ovarian bursa (OB) of PGCs aggregated with gonadal somatic cells. (*A*) Morphology of ovary like grafts at 45 days, 2 months and 3 months after transplantation (left). Follicles are not detectable in the sections by H&E staining following transplantation for longer-term (45 days, 2 months and 3 months) (right). The arrows indicate large vacuoles without follicles. (*B*) Co-immunostaining and fluorescence of DAZL and granulosa cell marker FOXL2. Scale bar =100 µm.

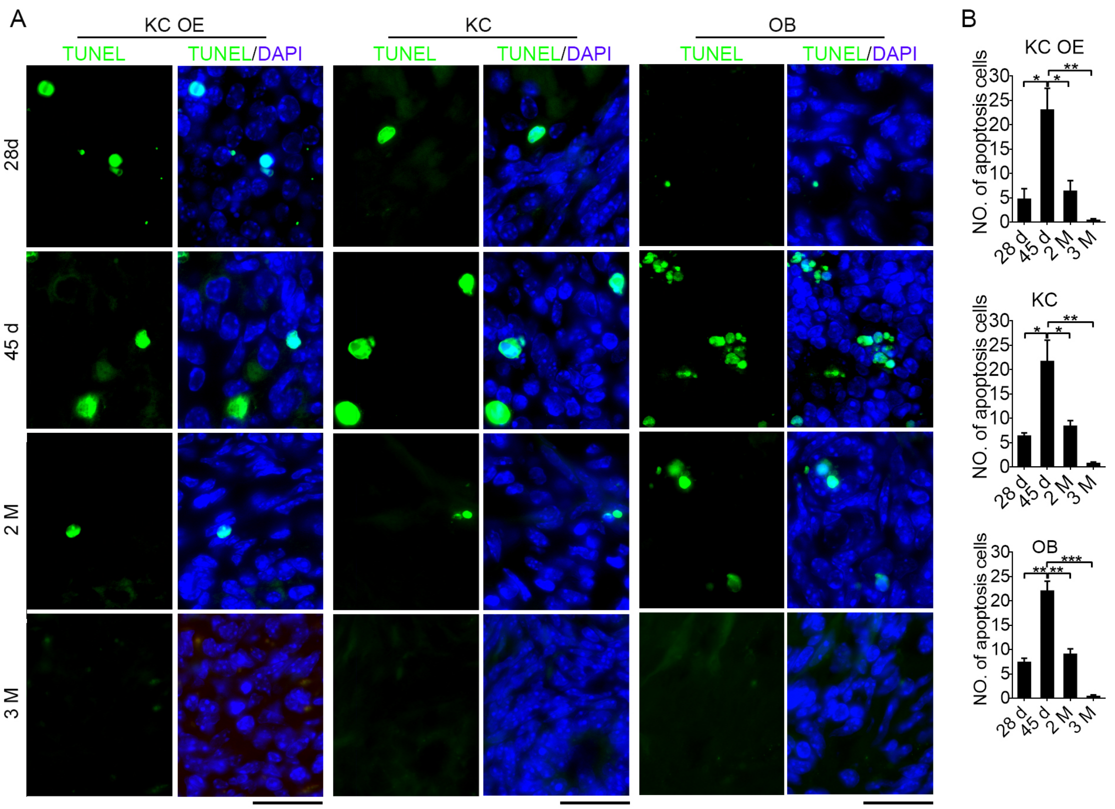

Fig. S6. Apoptosis by TUNEL assay of grafts by methods of KC OE, KC and OB. (*A*) Representative images showing apoptotic cells in the grafts 28 days, 45 days, and 2 and 3 months after transplantation. (*B*) Frequency of apoptotic cells per section in the graft originated by transplantation of PGCs-aggregates using 3 methods. Scale bar = 20 μm. Data represents Mean±SEM (n=3). *, P<0.05 ; **, P<0.01, ***, P<0.001.

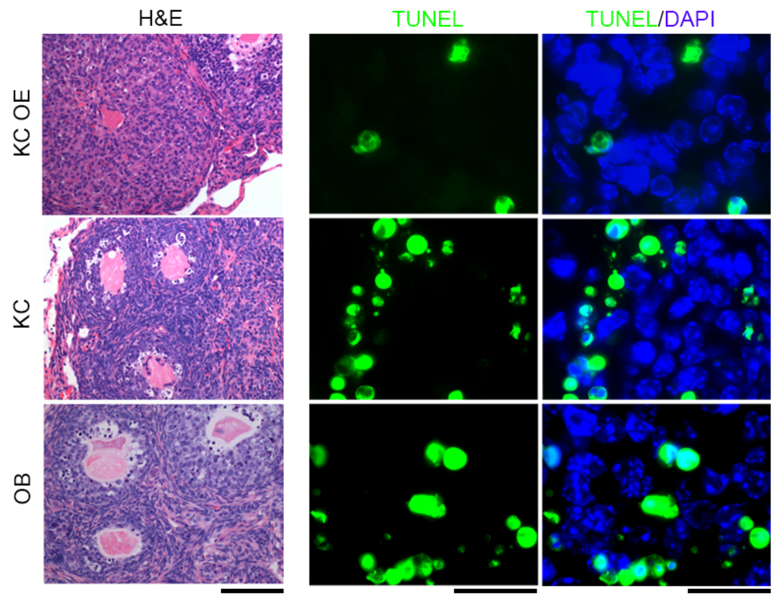

Fig. S7. Follicular atresia and oocyte degeneration and increased apoptosis in grafts at 32 days after transplantation by H&E staining (Left, Scale bar =100μm) and by TUNEL assay (Right, Scale bar = 20 μm).

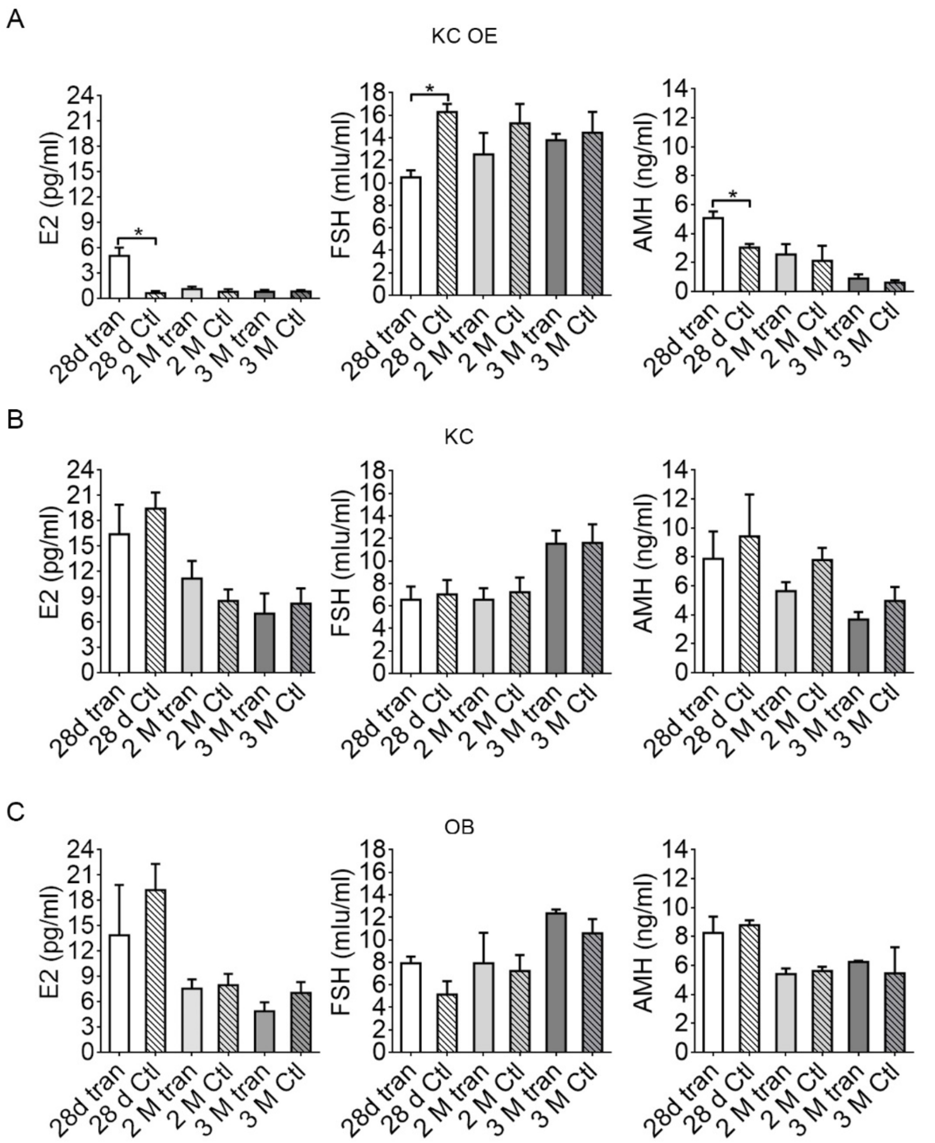

Fig. S8. Hormone levels in serum of the host animals grafted with PGCs-aggregates by three transplantation methods (*A,* KC OE; *B,* KC; *C,* OB). Serum of the host mice was collected 28 days, 2 and 3 months after transplantation, and E2, FSH and AMH levels measured as described in the Method. Shamed operation without transplantation of the cells served as control (Ctl) for each method. Data represents Mean ± SEM (n=3). *, P< 0.05.

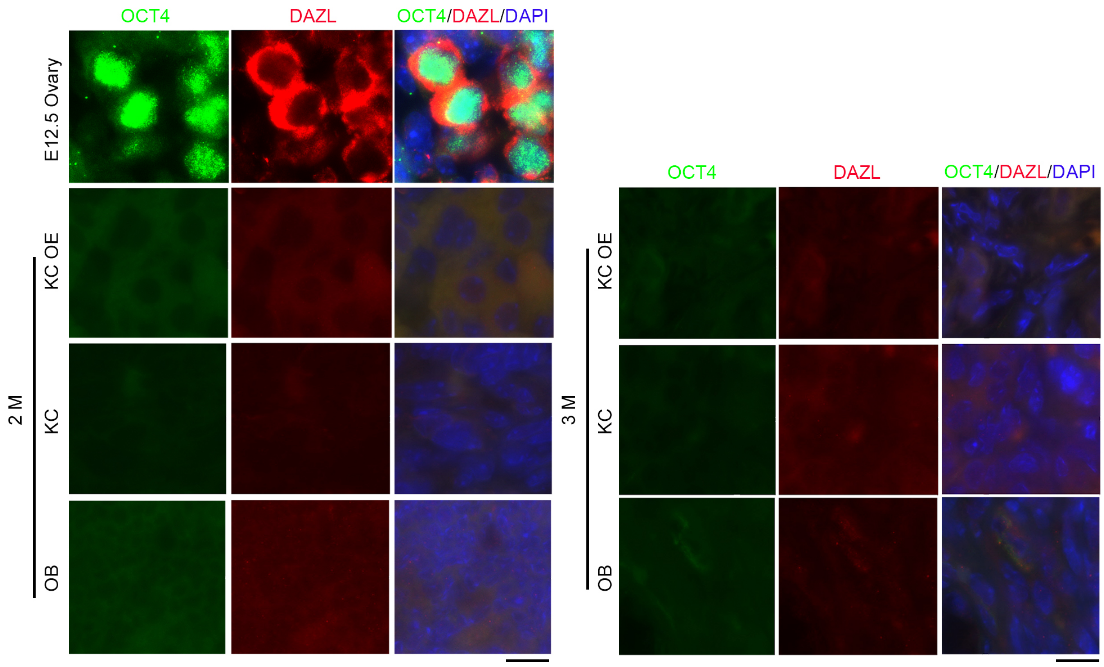

Fig. S9. Co-immunostaining and fluorescence microscopy of OCT4 and DAZL reveals germline stem cells (GSCs)/PGCs marked by nuclear OCT4 associated with cytoplasmic DAZL in E12.5 fetal ovaries, but not in the graft 2 and 3 months following transplantation of PGCs-aggregates. Scale bar = 10 μm.

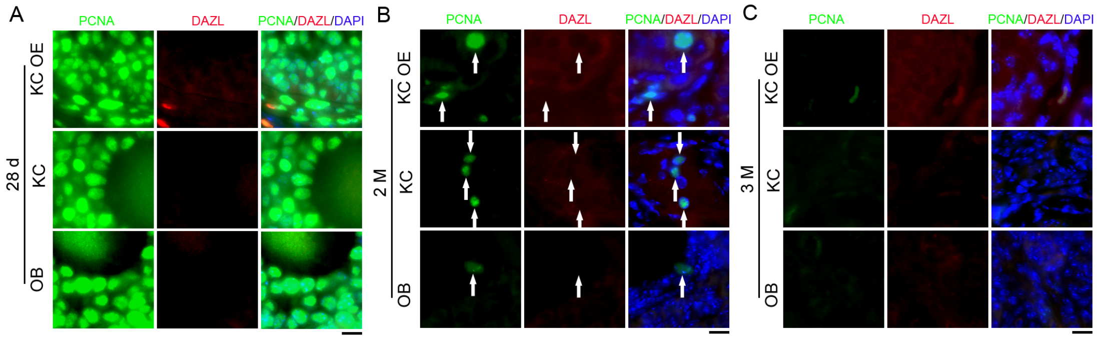

Fig. S10. Loss of proliferative female germ cells in 2 months and 3 months graft. Representative images showing co-immunostaining of PCNA and DAZL in grafts 28 days (*A*), 2 months (*B*) and 3 months (*C*) following transplantation. Granulosa cells in the follicles express high levels of PCNA and minimal DAZL in the graft after 28 days of transplantation. PCNA and DAZL double positive germ cells are not found in grafts (indicated by arrows shown in 2 months). Scale bar = 10 μm.

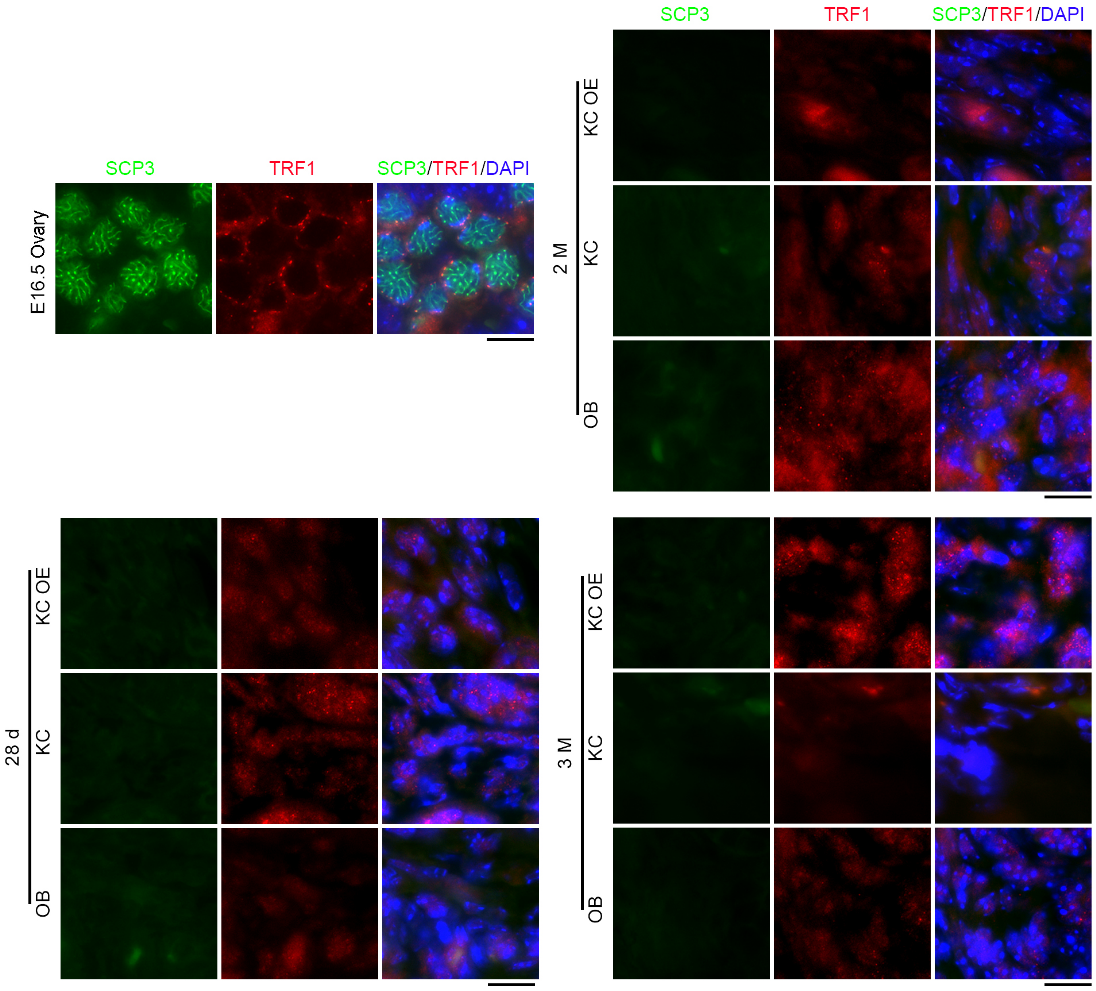

Fig. S11. Searching for neo-meiocytes in the grafts 28 days, 2 and 3 months following transplantation. Early meiocytes at prophase I in E16.5 fetal gonad displaying homologous pairing by SCP3 lateral filaments (green) with perinuclear TRF1 (red) at the termini are served as positive controls and these typical meiocytes could not be found in the grafts after 28 days. Scale bar = 10 µm.
